# Efficacy of Endolysin LysAB1245 Combined with Colistin as Adjunctive Therapy Against Colistin-Resistant Gram-Negative Bacteria

**DOI:** 10.3390/antibiotics14060538

**Published:** 2025-05-23

**Authors:** Rosesathorn Soontarach, Supayang Piyawan Voravuthikunchai, Potjanee Srimanote, Sarunyou Chusri

**Affiliations:** 1 Center of Antimicrobial Biomaterial Innovation-Southeast Asia, Faculty of Science, Prince of Songkla University, Songkhla 90110, Thailand; rosesathron_pim@hotmail.com (R.S.); supayang.v@psu.ac.th (S.P.V.); 2 Division of Infectious Diseases, Department of Internal Medicine, Faculty of Medicine, Prince of Songkla University, Songkhla 90110, Thailand; 3 Department of Medical Technology, School of Allied Health Sciences, Walailak University, Nakhon Si Thammarat 80161, Thailand; 4 Graduate in Biomedical Sciences, Faculty of Allied Health Sciences, Thammasat University, Pathum Thani 12121, Thailand; psrimanote01@yahoo.com.au

**Keywords:** antibiotic-resistant bacteria, endolysin, colistin, combination, antibacterial agent

## Abstract

**Background:** Colistin resistance among Gram-negative nosocomial pathogens is an increasing concern. The bacteriophage-encoded lytic enzyme endolysin LysAB1245, which targets bacterial peptidoglycan, was evaluated as a potential antibacterial agent in combination with colistin as a therapeutic approach. **Methods:** Clinical isolates of *Acinetobacter baumannii* and *Pseudomonas aeruginosa*, along with two reference strains, were used to assess the antibacterial activity of LysAB1245 and colistin, individually and in combination. Antibacterial susceptibility was assessed by broth microdilution. Synergistic interactions were determined using checkerboard assays and confirmed by time-kill kinetics. Resistance development was assessed after several rounds of exposure to each agent, either alone or in combination. **Results:** In this study, the synergistic activity of the LysAB1245/colistin combination therapy was found in some clinical isolates of *Acinetobacter baumannii* and *Pseudomonas aeruginosa*, resulting in a reduction in the MICs of both LysAB1245 and colistin. The bactericidal effects, with a significant, more than 3-log reduction in CFU/mL (*p* < 0.01), were observed in representative synergistic isolates within 4 h of treatment with the combination of LysAB1245 at 1/4 × MIC and colistin at 1/4 × MIC. Scanning electron microscope micrographs confirmed bacterial cell damage upon treatment with the combination. Additionally, treatment with LysAB1245 in combination with colistin had no effect on the development of bacterial resistance after multiple passages. **Conclusions:** Combining LysAB1245 with a last-resort antibiotic like polymyxins (colistin) could be used as a promising new antibacterial strategy for preventing and controlling antibiotic-resistant Gram-negative bacteria.

## 1. Introduction

The global prevalence of antibiotic-resistant bacteria in intensive care units (ICUs) has been steadily increasing [1,2,3]. Nosocomial infections are common among ICU patients, posing a public health threat and resulting in high rates of morbidity, mortality, and treatment cost [4,5,6]. Opportunistic Gram-negative bacteria, particularly *Acinetobacter baumannii* and *Pseudomonas aeruginosa*, are healthcare-associated pathogens commonly isolated from patients with ventilator-associated pneumonia, catheter-associated urinary tract infections, surgical site infections, and bacteremia [7,8,9,10]. Currently, both *A. baumannii* and *P. aeruginosa* have emerged as clinically significant pathogens, exhibiting resistance to widely used broad-spectrum antibiotics, including carbapenems, third-generation cephalosporins, and aminoglycosides [7,11,12,13]. Gram-negative bacteria employ diverse resistance mechanisms, notably the production of β-lactamases—hydrolytic enzymes that inactivate penicillin, monobactams, carbapenems, and cephalosporins [7,14,15]. Polymyxin E (colistin) is employed as a last-resort treatment for serious multidrug-resistant (MDR) Gram-negative infections, particularly those caused by *A. baumannii* and *P. aeruginosa*. However, its clinical utility is constrained by a high incidence of renal and neurological toxicity [16,17]. Moreover, the emergence of colistin-resistant Gram-negative bacteria has been documented in both developed and developing countries [18,19,20,21].

Due to the limited efficacy of conventional antibiotics, bacteriophages and their derivatives have been explored as alternative approaches for controlling drug-resistant infections [22]. One promising class of such agents is phage-derived peptidoglycan hydrolases, known as endolysins, which demonstrate potent antibacterial activity against antibiotic-resistant bacterial infections, including Gram-negative strains [23,24,25]. According to a previous study, LysAB1245,a purified endolysin derived from the *A. baumannii* phage T1245, has shown rapid and broad-spectrum lytic activity against multiple capsular types of MDR *A. baumannii* isolates [26,27]. Furthermore, LysAB1245 is a potential antibacterial agent with anti-biofilm properties for controlling both important Gram-negative and Gram-positive pathogens [28].

However, the development of endolysin-based therapeutics is currently associated with high costs, and prolonged monotherapy may foster bacterial resistance. This study investigated the synergistic effects of endolysin LysAB1245 in combination with commonly used antibiotics, such as colistin, against pathogenic Gram-negative bacteria. The aim was to enhance therapeutic efficacy, expand the antimicrobial spectrum, and reduce the effective dose requirements of each agent.

## 2. Results

### 2.1. Antibacterial Susceptibility Testing

The minimal inhibitory concentration (MIC) and minimal bactericidal concentration (MBC) values of endolysin LysAB1245 against all clinical isolates and reference strains ranged from 4.21 to 8.42 µg/mL (Table 1). Colistin exhibited antibacterial and bactericidal effects at concentrations of 1–4 µg/mL and 1–8 µg/mL, respectively. Based on MIC breakpoints, all tested isolates displayed a high or intermediate level of resistance to colistin.

### 2.2. Combined Antibacterial Activity of Endolysin LysAB1245 and Colistin

A checkerboard assay was conducted to investigate the combined effects of LysAB1245 and colistin on both *A. baumannii* and *P. aeruginosa* isolates (Table 2). Synergistic interactions were observed on *A. baumannii* (AB01 and AB04) and *P. aeruginosa* (PA04) clinical isolates, indicated by a four-fold reduction in the MICs of both agents. Additionally, the remaining isolates exhibited either additive or indifferent effects, and no antagonistic interactions were observed between LysAB1245 and colistin.

### 2.3. Time-Kill Kinetics Assay

**Figure 1:**
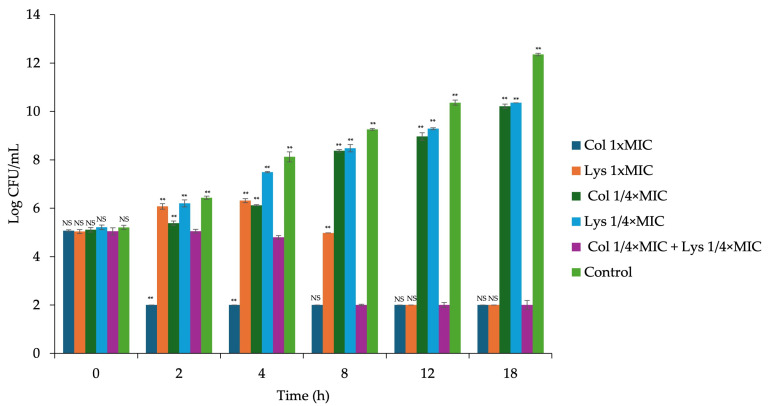
Time-kill curve of *Acinetobacter baumannii* AB01 isolate after treatment with colistin and endolysin LysAB1245, both individually and in combination, for 18 h. Results obtained from two independent experiments performed in triplicate are expressed as mean ± standard deviation (SD). Statistical analysis was compared with the combination, ** *p* < 0.01, and NS means non-significant.

Based on the checkerboard assay results, *A. baumannii* AB01 and *P. aeruginosa* PA04 were selected for time-kill analysis. Treatment with endolysin LysAB1245 (1/4 × MIC) combined with colistin (1/4 × MIC) exhibited synergistic effects on *A. baumannii* AB01, resulting in a significant reduction of more than 6-log CFU/mL at 8 h (*p* < 0.01), compared with the most active agent at the same concentration (colistin alone at 1/4 × MIC) (Figure 1). After 4 h of incubation, bactericidal activity was observed in *A. baumannii* AB01 cells exposed to colistin alone (1 × MIC) or the combination of LysAB1245 (1/4 × MIC) and colistin (1/4 × MIC), with more than a 3-log reduction in CFU/mL (99.9% viability), compared with the control, whereas treatment with LysAB1245 alone (1 × MIC) at 4 h demonstrated an approximate 2-log reduction in CFU/mL compared with the control.

**Figure 2:**
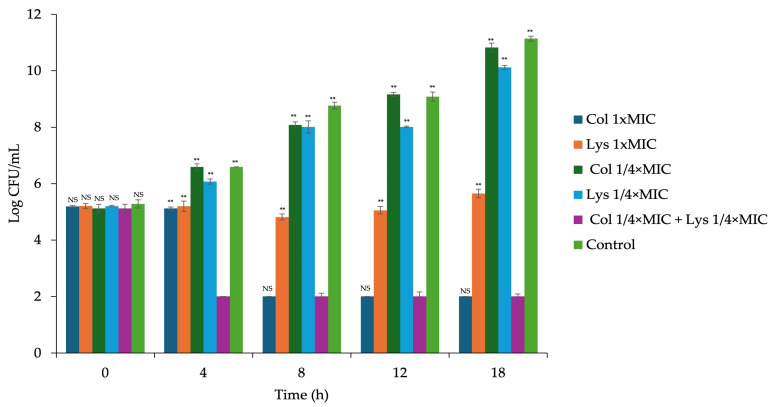
Time-kill curve of *Pseudomonas aeruginosa* PA04 isolate after treatment with colistin and endolysin LysAB1245, both individually and in combination, for 18 h. Results obtained from two independent experiments performed in triplicate are expressed as mean ± standard deviation (SD). Statistical analysis was compared with the combination, ** *p* < 0.01, and NS means non-significant.

For *P. aeruginosa* PA04, synergistic effects were observed, with a significant reduction of over 4-log CFU/mL in the viable bacterial count after 4 h of incubation with LysAB1245 (1/4 × MIC) combined with colistin (1/4 × MIC) (*p* < 0.01) (Figure 2). Furthermore, bactericidal effects of the combination were observed by a reduction of more than 4 log CFU/mL within 4 h, compared with the control.

### 2.4. Morphological Changes in A. baumannii AB01 and P. aeruginosa PA04 After Exposure to Endolysin LysAB1245 in Combination with Colistin

**Figure 3:**
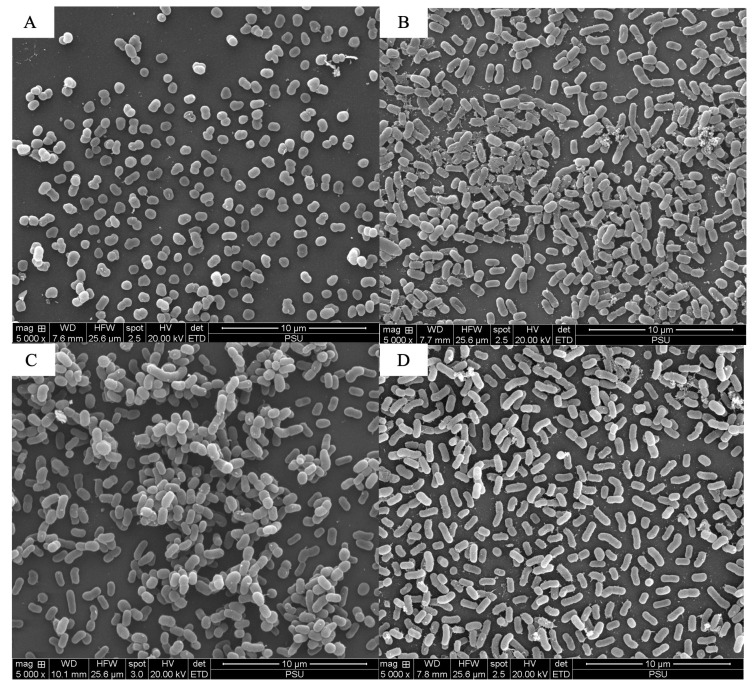
Scanning electron micrographs of *Acinetobacter baumannii* AB01 after exposure to LysAB1245 at 1/4 × MIC combined with colistin at 1/4 × MIC (**A**), LysAB1245 at 1/4 × MIC (**B**), colistin at 1/4 × MIC (**C**), and the control (**D**), all shown at a magnification of 5000×.

**Figure 4:**
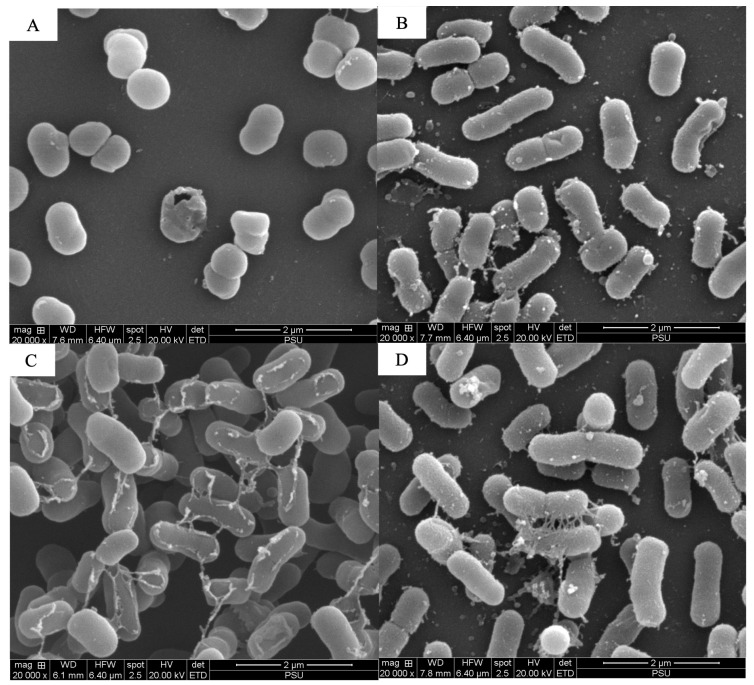
Scanning electron micrographs of *Acinetobacter baumannii* AB01 after exposure to LysAB1245 at 1/4 × MIC combined with colistin at 1/4 × MIC (**A**), LysAB1245 at 1/4 × MIC (**B**), colistin at 1/4 × MIC (**C**), and the control (**D**), all shown at a magnification of 20,000×.

**Figure 5:**
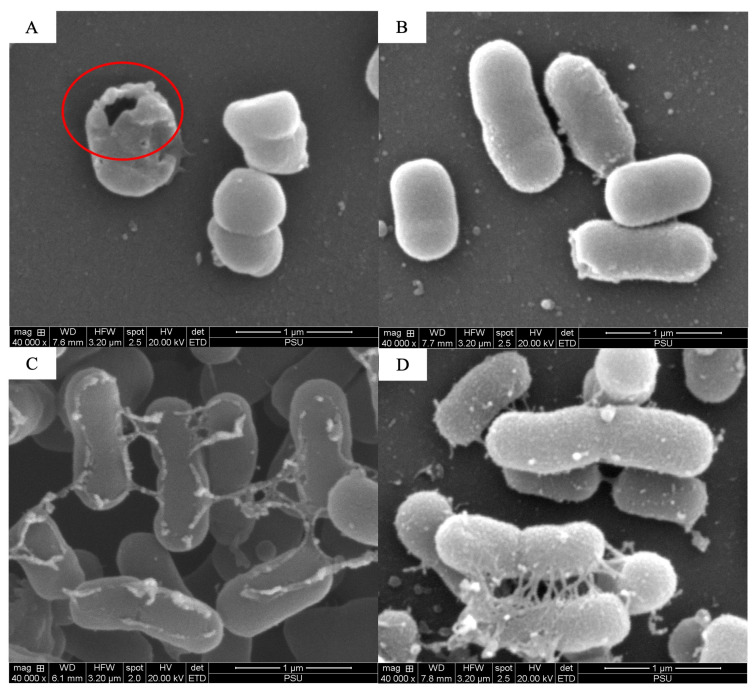
Scanning electron micrographs of *Acinetobacter baumannii* AB01 after exposure to LysAB1245 at 1/4 × MIC combined with colistin at 1/4 × MIC (**A**), LysAB1245 at 1/4 × MIC (**B**), colistin at 1/4 × MIC (**C**), and the control (**D**), all shown at a magnification of 40,000×. The presence of cell membrane damage is indicated by a circle symbol.

**Figure 6:**
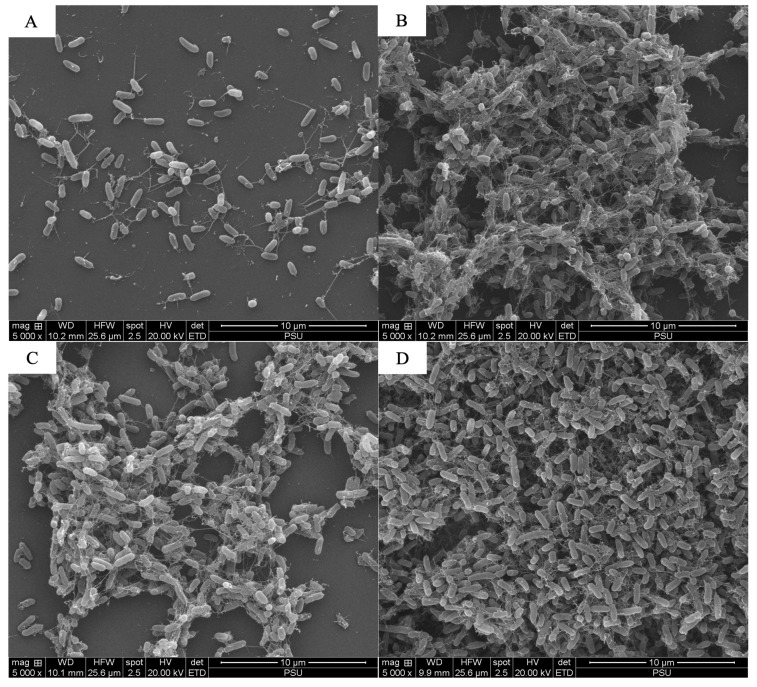
Scanning electron micrographs of *Pseudomonas aeruginosa* PA04 after exposure to LysAB1245 at 1/4 × MIC combined with colistin at 1/4 × MIC (**A**), LysAB1245 at 1/4 × MIC (**B**), colistin at 1/4 × MIC (**C**), and the control (**D**), all shown at a magnification of 5000×.

**Figure 7:**
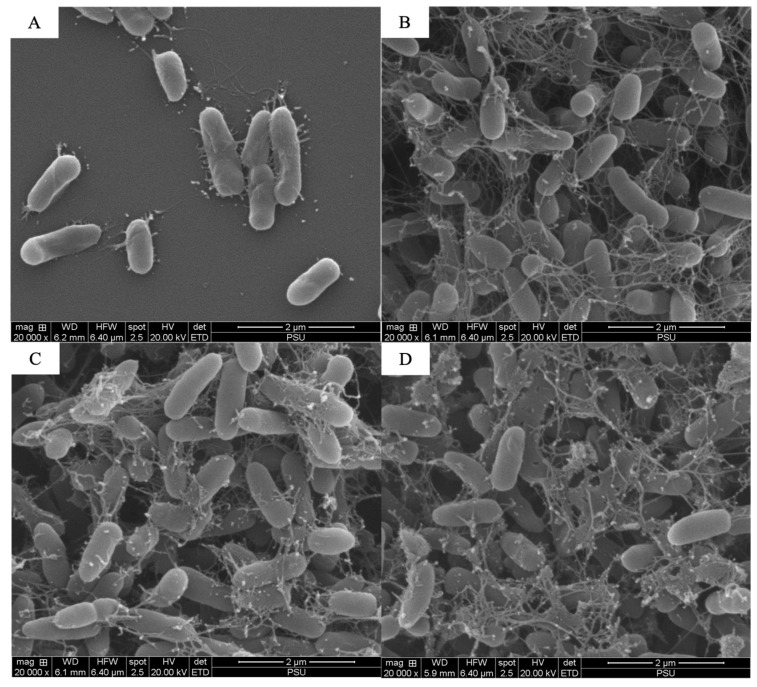
Scanning electron micrographs of *Pseudomonas aeruginosa* PA04 after exposure to LysAB1245 at 1/4 × MIC combined with colistin at 1/4 × MIC (**A**), LysAB1245 at 1/4 × MIC (**B**), colistin at 1/4 × MIC (**C**), and the control (**D**), all shown at a magnification of 20,000×.

**Figure 8:**
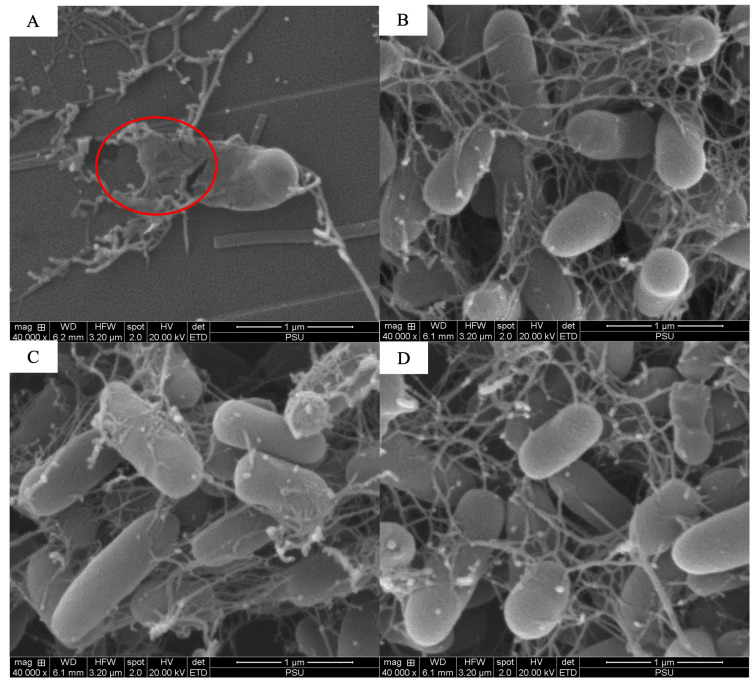
Scanning electron micrographs of *Pseudomonas aeruginosa* PA04 after exposure to LysAB1245 at 1/4 × MIC combined with colistin at 1/4 × MIC (**A**), LysAB1245 at 1/4 × MIC (**B**), colistin at 1/4 × MIC (**C**), and the control (**D**), all shown at a magnification of 40,000×. The presence of cell membrane damage is indicated by a circle symbol.

The scanning electron microscopy analysis revealed the combined effects of LysAB1245 (1/4 × MIC) and colistin (1/4 × MIC) on representative bacterial cells (Figure 3, Figure 4, Figure 5, Figure 6, Figure 7 and Figure 8). At a magnification of 40,000×, surface disruption and membrane damage were observed after 18 h of treatment (Figure 5A and Figure 8A). In contrast, treatment with LysAB1245 (1/4 × MIC) or colistin (1/4 × MIC) alone did not affect the surface morphology of either *A. baumannii* or *P. aeruginosa* cells (Figure 5B–D and Figure 8B–D). Additionally, the intact biofilm architecture was clearly observed on *P. aeruginosa* PA04 cells after treatment with each agent alone (Figure 6, Figure 7 and Figure 8).

### 2.5. Determination of Bacterial Resistance

Resistance development in *A. baumannii* and *P. aeruginosa* (clinical isolates and ATCC strains) after treatment with the combination of LysAB1245 and colistin, or each agent alone at 1/32 × MIC, was investigated (Table 3). The results indicated that the MIC values of LysAB1245 alone and in combination with colistin against all tested isolates remained stable across all conditions. After 10 rounds of exposure, the MICs of colistin alone were two-fold higher than the initial values for all isolates and returned to the initial levels after inoculation into an agent-free medium for both reference strains. The MICs of colistin when combined with LysAB1245 increased after exposure to the treatment but reverted to their baseline values after subsequent growth in an agent-free medium. Notably, the MICs of colistin in combination remained stable at 1 µg/mL for *A. baumannii* ATCC19606 and 0.5 µg/mL for *P. aeruginosa* ATCC27853 under all conditions.

## 3. Discussion

In 2024, the World Health Organization (WHO) classified carbapenem-resistant *A. baumannii* and *P. aeruginosa* as priority pathogens, highlighting the urgent need for research and the development of novel antibacterial agents [29]. Colistin, also known as polymyxin E, is considered a last-line treatment option for multidrug-resistant (MDR) and extensively drug-resistant (XDR) Gram-negative infections. Nevertheless, the global rise in colistin resistance in Gram-negative pathogens, particularly *Acinetobacter baumannii* and *Pseudomonas aeruginosa*, has been reported [30,31,32]. The high prevalence of colistin-resistant Gram-negative pathogens is often attributed to the loss or structural alteration of lipopolysaccharides (LPS), the primary target of colistin on the bacterial cell outer membrane [33,34]. Additionally, colistin treatment failure is often attributed to mobile colistin resistance mediated by plasmid-encoded mcr genes, as well as the activation of efflux pumps [21,35,36]. Importantly, colistin-induced pulmonary toxicity and nephrotoxicity are the most common side effects, which may limit its clinical use [37,38]. Therefore, the discovery and development of novel agents for controlling the emergence of antibiotic-resistant bacteria is a scientific challenge to the medical and scientific communities.

Bacteriophage-derived peptidoglycan hydrolases, called endolysins, have gained significant attention from research groups worldwide [39,40,41]. In a previous study, the endolysin LysAB1245 from *A. baumannii* phage was characterized and tested for its biological properties, demonstrating stability and a wide range of lytic effects on both Gram-negative and Gram-positive bacteria [27,28]. However, the production cost of purified endolysin is often high due to several factors. Furthermore, prolonged monotherapy can promote the development of bacterial resistance. Therefore, using adjuvant therapies in combination with endolysin to restore colistin efficacy is a promising approach to combating drug-resistant infections. The results of the combined effects revealed a synergistic activity against both *A. baumannii* and *P. aeruginosa* isolates, with a four-fold reduction in the MICs observed for LysAB1245 and colistin. Additionally, time-kill kinetics demonstrated bactericidal activity of the combination within 4 h of exposure. Recently, the interaction between endolysin and polymyxin E has been increasingly studied as a viable antimicrobial approach to combat antibiotic-resistant bacteria [42,43,44]. The combined activity of colistin and LysAB1245 can be attributed to the disruption of the LPS layer in Gram-negative bacteria, leading to increased membrane permeability. This action allows endolysin LysAB1245, a glycosidase hydrolase, to access the peptidoglycan layer, enhancing its ability to break down the bacterial cell wall. These complementary actions may enhance bacterial lysis while lowering the required colistin dose, thereby mitigating toxicity risks. In comparison with combinations such as β-lactam–polymyxin, the combination of endolysin LysAB1245 and colistin exhibits a distinct synergistic mechanism by integrating enzymatic degradation of the bacterial cell wall with membrane disruption. In contrast, traditional combinations primarily target membrane integrity or synthesis pathways, which may be less specific and carry greater risks of resistance development and adverse effects [45]. Scanning electron microscopy (SEM) images further confirmed the synergistic effects of LysAB1245 in combination with colistin on bacterial cell morphology, resulting in the rupture of the bacterial cell wall and cytoplasmic leakage. The combination also demonstrated effectiveness against strains of *P. aeruginosa*, which are often associated with biofilm production. Endolysins are recognized as potent anti-persister agents due to their ability to eliminate persister cells, which are typically tolerant to conventional antibiotics [46,47]. In this study, endolysin LysAB1245, encoded by the lytic *A. baumannii* phage T1245, demonstrated high intracellular stability, suggesting that continuous expression within the host strain does not compromise its viability. This finding implies a low probability of resistance development, a favorable attribute for long-term therapeutic use. Additionally, the MIC values of each agent in the combination remained stable across all conditions, suggesting that the combination of LysAB1245 and colistin could help prevent the emergence of colistin resistance in MDR pathogens. Notably, a previous study confirmed the antibacterial activity of combining endolysin ElyA1 and colistin in Galleria mellonella, as well as in murine skin and lung infection models [48]. This study has some limitations. First, it did not directly correlate molecular resistance mechanisms with patient-level outcomes, such as treatment failure, morbidity, or mortality. Second, many insights into resistance mechanisms are based on in vitro models, which may not accurately replicate host–pathogen interactions, immune responses, or pharmacokinetics in clinical settings. Animal models and clinical studies are required to validate the clinical relevance of in vitro resistance findings and better predict treatment outcomes.

## 4. Materials and Methods

### 4.1. Bacterial Strains and Culture Conditions

Four clinical isolates each of *A. baumannii* and *P. aeruginosa* were previously collected from patients in Songklanagarind Hospital (Ethical Approval No. REC 59-241-19-6). The standard reference strains, *A. baumannii* ATCC 19606 and *P. aeruginosa* ATCC 27853, were used in this study. All isolates were cultured at 37 °C in Tryptic soy broth (TSB) (Difco Laboratories, Detroit, MI, USA) and subcultured on Tryptic soy agar (TSA, Difco). Bacterial stocks were kept at −80 °C in TSB supplemented with 20% (*w*/*v*) glycerol.

### 4.2. Antibiotic Susceptibility

Purified endolysin LysAB1245 was sourced from a previous study [27]. The MIC values of purified endolysin LysAB1245 and colistin (Thermo Fisher Scientific, Waltham, MA, USA) were determined against eight clinical isolates and two reference strains of *A. baumannii* and *P. aeruginosa* using the broth microdilution method, following Clinical and Laboratory Standards guidelines [49]. In brief, bacterial cultures were grown to logarithmic phase in Mueller–Hinton broth (MHB, Difco) at 37 °C with shaking at 150 rpm.

After that, 50 μL of bacterial suspension at 10^6^ CFU/mL was added to the well containing 50 μL of a 2-fold diluted preparation of LysAB1245 or colistin in MHB, and the polystyrene 96-well plate was further incubated at 37 °C for 16–18 h. Subsequently, 10 μL of resazurin solution (0.01%) was added to all wells and further incubated at 37 °C for 2 h. The MIC was defined as the lowest drug concentration that inhibited bacterial growth, while the MBC was determined by the absence of colony growth from direct well contents. Antibiotic susceptibility was interpreted according to CLSI breakpoints: intermediate (2 μg/mL) and resistant (≥4 μg/mL). The experiment was performed in triplicate for two independent repeats.

### 4.3. Checkerboard Synergy Testing

The effects of combining LysAB1245 and colistin on the eight clinical isolates and two ATCC strains were evaluated using a checkerboard assay, as described in a previous study [50]. Briefly, log-phase bacterial cultures were prepared in MHB and adjusted to 10^6^ CFU/mL. Endolysin LysAB1245 was serially diluted two-fold in a vertical orientation, while colistin was diluted two-fold horizontally (ranging from 1/16 MIC to 2 MIC) in a 96-well microplate, with a final volume of 50 μL per well. After that, 50 μL of bacterial cultures was gently added to each well of a 96-well plate containing diluted combined agents. Bacteria incubated with MHB or each agent alone was used as a control. After incubation at 37 °C for 18 h, the fractional inhibitory concentration index (FICI) was calculated by the following equation:

$$\mathrm{FICI}=\frac{\mathrm{MIC}\;\mathrm{of}\;\mathrm{drug}\;A\;\mathrm{in}\;\mathrm{combination}}{\mathrm{MIC}\;\mathrm{of}\;\mathrm{drug}\;A\;\mathrm{alone}}+\frac{\mathrm{MIC}\;\mathrm{of}\;\mathrm{drug}\;B\;\mathrm{in}\;\mathrm{combination}}{\mathrm{MIC}\;\mathrm{of}\;\mathrm{drug}\;B\;\mathrm{alone}}$$

The effects of the tested combination were interpreted based on the following cutoffs: FICI ≤ 0.5, synergistic; 0.5 < FICI ≤ 1.0, additive effect; 1.0 < FICI ≤ 4.0, indifference; and FICI > 4.0, antagonistic [50]. The experiment was performed in triplicate for two independent repeats.

### 4.4. Time-Kill Curve Assays

The combined activity of colistin and LysAB1245 against representative *A. baumannii* and *P. aeruginosa* clinical isolates was confirmed using a time-kill kinetics assay, following the method described by Díez-Aguilar et al. [51] with slight modifications. Briefly, the selected isolate was grown to the log phase in MHB, and the initial bacterial inoculum was adjusted to 106 CFU/mL. The cultures were then added to tubes containing a combination of LysAB1245 and colistin at different concentrations and incubated at 37 °C. Samples were obtained at 0, 2, 4, 6, 8, 12, and 24 h and serially diluted 10-fold in 0.85% sterile saline for colony counting. Bacteria incubated with MHB, or each agent alone, were used as controls. Time-kill curves were generated by plotting the logarithmic number of bacterial cell viability against incubation time. Synergy between the two agents was defined as a 2-log reduction in CFU/mL compared with the most effective agent used alone, which signifies a significant increase in bactericidal activity beyond that of each agent individually. A reduction of less than 2-log CFU/mL was considered indifference, whereas antagonism was defined as a 2-log increase in CFU/mL compared with the most active single agent (CLSI, 2023 [49]). Bactericidal activity was defined as a 3-log reduction in CFU/mL, while bacteriostatic activity was defined as a reduction of less than 3 log CFU/mL compared to the control. Limit of detection (LOD) for surviving bacterial cells was 2 log CFU/mL. Experiments were performed in triplicate with two independent repeats. Statistical analyses between the combination and other test groups (each agent alone and the control) were performed using GraphPad Prism v10.2.3 Comparisons between means were carried out according to Tukey’s multiple comparison test at 99% confidence interval.

### 4.5. Scanning Electron Micrographs of A. baumannii AB01 and P. aeruginosa PA04

#### After Exposure to LysAB1245 Combined with Colistin

The effects of the combination on bacterial cell morphology were observed via SEM. Briefly, a log-phase bacterial culture was adjusted to 106 CFU/mL and added into a tube containing LysAB1245 (1/4 × MIC) in combination with colistin (1/4 × MIC). The mixture of bacterial cells and MHB or each agent alone served as controls. After incubation at 37 °C for 18 h, bacterial pellets were harvested by centrifugation at 10,000× *g* for 5 min, washed twice with 10 mM phosphate-buffered saline (PBS, pH 7.4), fixed in 2.5% (*w*/*v*) glutaraldehyde (Sigma-Aldrich, St. Louis, MO, USA), and dehydrated through a graded ethanol series (20–99.9%). Samples were then subjected to critical point drying, gold coating, and examination using a scanning electron microscope (SEM; Quanta 400 FEG, FEI, Hillsboro, OR, USA).

### 4.6. Resistance Development Determinations

A broth microdilution method was used to observe the development of resistance in bacterial cells during treatment with LysAB1245 combined with colistin, as previously described with some modifications [52]. Briefly, representative isolates of *A. baumannii* AB01 and *P. aeruginosa* PA04 (106 CFU/mL) were exposed to LysAB1245 (1/32 × MIC) in combined with colistin (1/32 × MIC) or each agent alone (1/32 × MIC) and incubated at 37 °C for 18 h. Next, the mixtures with bacterial growth were inoculated into fresh TSB (1:100) for the next round of exposure. After 10 rounds of exposure, cultures were inoculated into fresh TSB without treatments for an additional five rounds, allowing for the potential reversion of the bacterial phenotype to its original, non-resistant state. After that, the cultures were streaked on MHA plates and subsequently subjected to MIC determination.

## 5. Conclusions

This study demonstrated that endolysin LysAB1245, when combined with polymyxin E (colistin), is an effective strategy against drug-resistant Gram-negative bacterial pathogens. The combination not only enhanced antibacterial activity but also reduced the required dose, thereby minimizing the risk of side effects associated with high antibiotic concentrations. Moreover, no resistance development was observed during treatment, highlighting the potential of this synergistic approach in combating drug-resistant infections.

## Figures and Tables

**Table 1:** Minimal inhibitory concentrations (MICs) and minimal bactericidal concentrations (MBCs) of endolysin LysAB1245 and colistin against *Acinetobacter baumannii* and *Pseudomonas aeruginosa* isolates.

Tested Organisms	Values of MIC/MBC (µg/mL)		Interpretation
	LysAB1245	Colistin	
*A. baumannii*			
AB01	4.21/4.21	4/4	Resistant
AB02	4.21/4.21	2/4	Intermediate
AB03	8.42/8.42	4/8	Resistant
AB04	4.21/4.21	4/4	Resistant
ATCC19606	4.21/4.21	1/1	Intermediate
*P. aeruginosa*			
PA01	8.42/8.42	4/8	Resistant
PA02	4.21/4.21	2/2	Intermediate
PA03	8.42/8.42	4/4	Resistant
PA04	4.21/8.42	4/4	Resistant
ATCC27853	4.21/4.21	1/1	Intermediate

**Table 2:** Minimal inhibitory concentration (MIC) values and fractional inhibitory concentration (FIC) indices of endolysin LysAB1245 and colistin, alone and in combinations, against *Acinetobacter baumannii* and *Pseudomonas aeruginosa* isolates, as determined by checkerboard assay.

Isolates	MIC (µg/mL)				ΣFIC	Interpretation
	LysAB1245	Colistin	LysAB1245 in Combination	Colistin in Combination		
*A. baumannii*						
AB01	4.21	4	1.05	1	0.5	Synergistic
AB02	4.21	2	2.10	1	1	Additive
AB03	8.42	4	4.21	0.5	0.63	Additive
AB04	4.21	4	1.05	1	0.5	Synergistic
ATCC19606	4.21	1	4.21	1	2	Indifferent
*P. aeruginosa*						
PA01	8.42	4	4.21	2	1	Additive
PA02	4.21	2	4.21	2	2	Indifferent
PA03	8.42	4	1.05	2	0.62	Additive
PA04	4.21	4	1.05	1	0.5	Synergistic
ATCC27853	4.21	1	2.1	0.5	1	Additive

**Table 3:** Stepwise selection of *Acinetobacter baumannii* and *Pseudomonas aeruginosa* cells after treatment with endolysin LysAB1245 and colistin alone and in combinations.

Isolates	Treatments	MIC (µg/mL)		
		Initial	After 10 Rounds of Exposure	After Inoculation into an Agent-Free Medium
*A. baumannii*				
AB01	LysAB1245	4.21	4.21	4.21
	Colistin	4	8	8
	LysAB1245 in combination	1.05	1.05	1.05
	Colistin in combination	1	2	1
ATCC19606	LysAB1245	4.21	4.21	4.21
	Colistin	1	2	1
	LysAB1245 in combination	4.21	4.21	4.21
	Colistin in combination	1	1	1
*P. aeruginosa*				
PA04	LysAB1245	4.21	4.21	4.21
	Colistin	4	8	8
	LysAB1245 in combination	1.05	1.05	1.05
	Colistin in combination	1	2	1
ATCC27853	LysAB1245	4.21	4.21	4.21
	Colistin	1	2	1
	LysAB1245 in combination	2.1	2.1	2.1
	Colistin in combination	0.5	0.5	0.5

## Data Availability

Data are available in a publicly accessible repository and within the article.
